# On-Body Placement of Wearable Safety Promotion Devices Based on Wireless Communication for Construction Workers-on-Foot: State-of-the-Art Review

**DOI:** 10.3390/s22093134

**Published:** 2022-04-20

**Authors:** Neeraj Yadav, Neda Sadeghi, Julian Kang

**Affiliations:** 1 Department of Architecture, 3137 TAMU, College Station, TX 77843, USA; 2 Department of Construction Science, 3137 TAMU, College Station, TX 77843, USA; nsadeghi@tamu.edu (N.S.); juliankang@tamu.edu (J.K.)

**Keywords:** construction safety, wearable safety promotion devices, workers-on-foot, personal protective equipment, communication network, on-body placement

## Abstract

High auditory noise levels and limited visibility are often considered among the main factors that hinder seamless communication on construction sites. Many previous research studies have leveraged technology to overcome these obstacles and communicate using the hearing, sight and touch senses. However, the technological efficacy does not secure the users’ perceptivity of the wireless communication devices. Statistical data regarding the number of fatal accidents on construction sites have remained steady despite regular efforts. This study analyzed prior research on wearable safety promotion devices for personnel that move around the jobsite on foot. A seven-point checklist was utilized to shortlist prior studies (2005–2021) attempting to provide safety information wirelessly to the construction workers-on-foot. The reasoning behind various on-body placements was investigated along with the information conveyed using the three communication modalities. A novel communication network is also introduced to visualize the technical details. Lastly, limitations and future recommendations have been presented to gain insights about the factors that might affect the placement of the wearable safety promotion devices.

## 1. Introduction

Construction has been ongoing since the inception of human civilization. Many structures that were built centuries ago still stand to date, including some complex undertakings, which makes us marvel at their existence. However, there is limited documentation, if any, of the building process, working conditions or casualties involved in those projects. In today’s modern world, the oversight of judicial authority, government and non-profit organizations, as well as the reach of communication media, has resulted in a fair amount of documentation regarding any untoward incident [1]. While most projects start out to be fairly organized, the work environment tends to get more complex as the construction progresses, with many contractors and subcontractors aiming to meet the target completion dates. There is little room for error since the work happens in close proximity to other unfamiliar professionals and heavy equipment along with some dangerous working conditions resulting from the very nature of this profession [2,3].

However, ensuring the health and safety of the skilled construction workforce is critical. Occupational Safety and Health Administration (OSHA), the regulatory body in establishing workplace safety, has mandated the use of Personal Protective Equipment (PPE) along with safety training and education to increase safety and awareness on construction jobsites [4]. However, continuous improvement becomes significant for the construction industry in order to keep its current workforce safe and healthy, and to best reach and attract the future generation for careers in construction.

### 1.1. Fatal Accidents on Construction Sites

Every single life lost at work is one too many. Numerous technological attempts have been made to reduce accidents and injuries on construction jobsites with partial success. However, recent reports still indicate the construction industry to be one of the most fatal workplace environments. The recent five-year data (2014–2018) of fatal injuries, released by the Bureau of Labor Statistics (BLS), U.S. Department of Labor, were interpreted to break down the major areas of concern in construction [5]. During those five years, the industry was responsible for the loss of 4806 lives, which accounted for over 19 percent of the total fatalities across all industries. Therefore, it can be assumed that the measures undertaken by the construction industry and the regulatory authorities over the past several decades are not complete.

**Figure 1:**
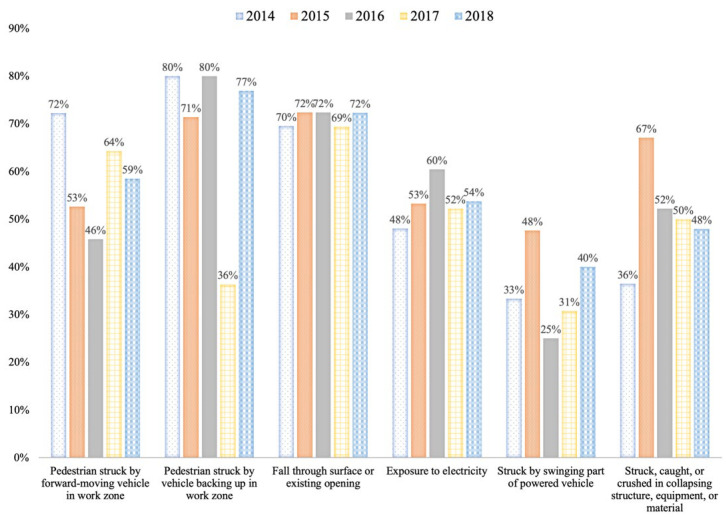
Certain events in the US construction industry have a very high fatality rate as compared to the yearly total fatalities occurring across all sectors [5].

Nearly one out of every five lives lost from workplace injuries was in the construction industry. However, the situation is even more grim when considering fatal pedestrian exposure to vehicles or fall-through openings, where the construction industry accounts for over three out of five fatalities. Figure 1 displays some of the events and exposures which are responsible for a high amount of yearly fatal injuries in the construction industry as compared to the rest of the industries.

Referring to Figure 1 again, the fatal accidents due to the pedestrians being struck by a vehicle, both forward-moving and backing up, have failed to reach a steady decline. Meanwhile, the fatal accidents related to falling through the surface or existing openings have seen a negative change similar to the rate of accidents regarding exposure to electricity. Fatal injuries resulting from being stuck by the swinging part of a powered vehicle and being stuck, caught or crushed in collapsing structure, equipment or material have also failed to show any consistent decline. Cumulatively, they were responsible for 1074 out of 1876 fatalities across all industries between 2014 and 2018. Additionally, since a majority of these fatalities are in construction, the industry cannot look to any other industry for potential solutions to these events.

The construction work environment certainly contributed to these injuries and there is a need to address factors that might be absent in other industry environments [6,7]. Since the workers-on-foot (that is the workers navigating the construction jobsite on foot) are generally expected to be responsible for their own safety, we can make reasonable assumptions that they were hit by vehicles because they did not know a certain vehicle or equipment was approaching them. If they had visually recognized the vehicle approaching them, then they would have kept a certain distance from them for their own safety. If they did not visually recognize the vehicle approaching them, they should have noticed the noise or audible alarms from the vehicle approaching them. If they were hit by a vehicle approaching in a direction they did not see visually, we can reasonably assume that the noise from the vehicle gave them little warning. Why did they not hear the noise from the vehicles approaching them? There may be several reasons for this, but one of them could be related to the auditory noise levels on construction sites.

### 1.2. Auditory Noise on Construction Sites

Construction jobsites have been well documented to have high auditory noise levels due to the constant use of machinery and equipment. The average noise levels can reach 80–90 decibels, while in some instances it can go up to 125 decibels, making the occupational noise exposure in construction hazardous [8,9,10]. Hence, it is very likely that the sound produced by an average person’s yell or a loud alarm on the construction site is not a particularly noticeable sound. In many situations, the field personnel can be prohibited from collecting any information by sound which can cause them to be unaware of a vehicle approaching them.

Noise related to machinery and equipment is also a significant issue in the military. However, the efforts by the U.S. Department of Defense to counter the high auditory noise levels are noteworthy since their regulations tend to be conservative as compared to the OSHA standards [11,12]. Additionally, the equipment manufacturers are required to dedicate adequate attention to noise reduction mechanisms and acoustic insulations. The construction industry should certainly try to emulate those design criteria for limiting the noise levels on the jobsite.

Prior studies have consistently reported that high auditory noise is a significant issue that contributes to construction site accidents [13,14]. While this has certainly played a role in injuries involving heavy vehicles, the high amount of fatalities resulting from fall through surface or existing openings to lower levels likely involves other factors.

### 1.3. Visual Hinderance on Construction Sites

Assuming the personnel did not lose their balance or actively step onto the existing surface openings, a reasonable explanation for falls to lower levels could be due to some form of visual hinderance to the line of sight of the construction personnel. The dynamic nature of construction also contributes to the spatial and temporal safety risks [15]. Additionally, there are many possible reasons a potential hazard cannot be identified in a timely manner due to visual hinderance. The weather certainly plays a major role in determining the visibility of any given location, and its role in accidents is also well documented. For instance, a construction personnel working on a roof fell through an existing opening that was covered with snow [16]. Factors like fog and rain can also severely impact the identification of any potential hazard or protective reflective clothing due to limited visibility.

The line of sight could also be impacted by the presence of equipment or other temporary installations. Meanwhile, the communication between different work levels is also somewhat restricted due to the built environment itself. It is difficult for a person to reach out or visually signal something to another person working beyond a certain distance, either horizontally or vertically, if they cannot establish an initial eye contact. Similarly, any information to be conveyed through visual alerts or warning signs is only effective after establishing the initial eye contact.

Lighting in the work environment also impacts the visual information that can be gathered by the construction professionals. While an outdoor job in daylight might not have any significant issues, some of the interior temporary work zones might not be well lit enough to identify a potential hazard or to make a well-informed assessment. Conversely, if the interior area lighting is not set up correctly, the strong glare from the lights might contribute to reduction in information that can be taken in as compared to an adequately lit environment. Aside from the auditory and visual obstructions, there are other factors that can potentially hamper communication on construction sites.

### 1.4. Other Hinderances on Construction Sites

While limitations and breakdown in visual and auditory information play a major role in construction fatalities, other sensory impairments can also increase the likelihood of an occupational injury. For instance, olfactory noise such as odor from concrete, dust, other construction waste or standing water from rain or excavation can override the smell of chemicals such as toxic gas leaks or smoke. Meanwhile, vibration noise from equipment like heavy vehicles or jackhammer can impact the operators’ ability to sense vibration alerts.

The importance of alerting construction workers gains further significance because studies have shown that fatigue and task repetition results in lower awareness, performance and loss of focus [17,18,19]. Jobsite congestion, especially during the later stages of a project, can also impact the personnel safety. New studies are being conducted to better understand situational awareness in hazardous conditions [20]. Meanwhile, it is known that equipment operators in mental overload are significantly hampered in their ability to make safe decisions [21]. Another possibility is that since construction work usually goes on simultaneously on multiple levels with similar floor plans, it is possible to mistakenly assume a sense of security based on certain confidence of familiarity with a different level.

Language barrier can be considered as another significant factor which hinders smooth communication between construction personnel. As many of the foreign construction personnel cannot speak or understand the local language, miscommunication caused by this language difference creates significant problems during construction tasks, and limits the information that can be conveyed [22].

### 1.5. Early Attempts at Overcoming Challenges

Various methods have been tried to overcome these obstacles that hinder communication on construction sites. Hand signals are a long-standing means of communication between field workers on noisy construction sites. However, hand signals require a line of sight, and expressions that can be transmitted through hand signals are limited [23]. Other regularly implemented solutions include passive information displays such as hazard labels and placement of physical barriers including cautionary tapes and cones.

Walkie-talkie is one of the prominent means of actively overcoming communication barriers. It enables field personnel to communicate with each other without being constrained by distance, noise and line of sight [24]. However, walkie-talkie requires active responses from users and communication does not take place unless the user picks up the communication channel. This can drastically hinder smooth communication in urgent situations.

Unlike walkie-talkies, wireless data communication has opened up the possibility of sending and receiving information without requiring an active response from the receiver. This seamless communication is made possible by attaching portable tags and electronic sensors to users’ body.

## 2. Construction Site Safety Enhancement Efforts

Several previous research studies have made contributions to enhance safety on construction sites, but many limitations and barriers continue to be documented [25,26]. A majority of the prior efforts could be largely classified into two categories. The first category includes assessing the situation by collecting information wirelessly from the construction personnel and accurately predicting their condition at that instance. The second category includes wireless communication to the construction personnel of any potential or impending hazard.

### 2.1. Safety Monitoring Using Wearable Sensing Devices

Regarding the assessment and prediction of the present condition of the construction personnel, the raw data collected using wireless communication is usually in form of the location, the voluntary physical behavior or the involuntary physiological condition of the personnel. This would not have been possible without the advancements in various sensing technologies and their applications towards enhancing safety and health on construction sites [27,28,29,30,31,32,33,34,35,36,37,38].

The location or position data refers to the presence of construction personnel in a workspace. It is often collected to assess if the field personnel are in proximity of any potentially dangerous equipment or environment. This could either be in terms of the relative distance between the personnel and the objects of interest [39,40] or in terms of the absolute position of the personnel on a local [41] or global coordinate system [42,43]. Additionally, work is being conducted towards prediction of trajectories based on sensor data [44].

Aside from the location or position data, the voluntary physical behavior concerns the physical actions that can be controlled by the construction personnel with relative ease, for instance, physical posture or eye gaze. One potential use is to predict injury inducing posture [45]. It can also be used to assess fall risks as carried out by placing accelerometers on certain body parts of the construction personnel such as the hard hat [46], waistline [47,48], ankle [49] or across the body [50].

The involuntary physiological condition of the personnel refers to the body’s internal indicators such as body temperature, pulse, oxygen saturation, electroencephalography (EEG), pupil changes among others [51,52,53,54]. Research is also ongoing with regards to collecting data from pressure sensors attached to various body parts such as the shoe soles [55]. Any anomaly or deviation from previously validated inputs can imply unsafe behavior and can help understand factors like physical health, fatigue and focus of the construction personnel. This can support monitoring the well-being of the personnel while passively analyzing the behavior, productivity and safety issues.

### 2.2. Efforts Directed towards Safety Warnings

The second category of prior efforts is related to the use of wireless data communication to actively inform the construction personnel about any potential or impending hazard. This transfer of information to the construction personnel of any perceived risks, communicated wirelessly in a timely manner, often relies on the human hearing, sight and/or touch sense. Such communication could be achieved with the help of portable devices that could either be worn on the body or held-in-hand by the construction field personnel.

Another possibility is the strategic activation of alert devices placed in the construction environment [56,57,58,59,60], including the prospect of embedding the alerts in smart tools [61,62,63] or to convey feedback and alerts to the managerial supervisory team through dashboards and personalized texts or emails [64,65,66,67,68]. In one instance, a pulse oximetry sensor was integrated into a hard hat to protect construction workers from carbon monoxide poisoning [51]. It mentioned the vision for a wide alert system that can warn co-workers and supervisors with visual and audible cues to the location of the worker.

Another study explored sending text messages to the safety supervisors and pop-up alarms on computer screens in addition to alarms placed at the hazardous sites [69]. The zone alarms increase in intensity and volume if the worker is nearing the hazard. Meanwhile, in another instance, graphical user interface (GUI) alerts were sent to the safety supervisor in addition to the alerts for the equipment operators [70]. Communicating alerts through a portable device attached onto the equipment or communicating the alerts directly to the equipment operator has been a fairly well-explored field [41,71,72,73,74,75,76].

The idea of using a wearable device to warn the workers-on-foot about equipment proximity has been around for a while. In one study, the authors envisioned such a device to be worn at the waist belt, and configured the handheld receiver to include auditory and tactile alerts but no visual indicator [77]. Another study attempted to reduce pedestrian-vehicle collisions by presenting a conceptual model SightSafety with graduated audio tone depending on the level of danger [78]. However, the on-body placement of the wearable micro-electro-mechanical systems (MEMS) tag was not discussed.

The use of wireless local area network (WLAN) and global positioning system (GPS) has also been explored for construction sites. One such study utilized it for ubiquitous location tracking and delivery of context-specific visual information to the head mounted display of the wearer, with construction safety being a potential application [79]. Meanwhile, the use of wearable tags has also been explored in prior literature for work zone safety. However, many studies have used them either specifically for information about positioning and localization [80,81] or utilized such information to provide handheld visual, auditory or vibratory alerts [40,82,83,84,85,86,87,88,89,90,91].

One study conducted at the National Institute for Occupational Safety and Health (NIOSH) evaluated the degrading effect of mechanical vibrations at the feet on balance [92]. The study was conducted in a virtual reality system that simulated a narrow plank at elevation on a construction site and compared three states—no vibration, sub threshold vibration and supra threshold vibration. The study participants stood on an instrumented gel insole with vibrating tactors in standard posture and semi-tandem posture. The insoles were not designed to be inserted in the shoes and were not attached to the feet of the participants.

Another study made use of Fiber Bragg Granting sensor-based radio frequency locating system to communicate warnings through portable tags mounted on the safety suit to convey the work environment and structure safety status on an underground metro tunnel project [56]. However, the study failed to specify the exact on-body placement of the portable tag on the safety suit to convey warning lights and alarm bells. Another study evaluated workers’ responses to auditory proximity warnings with 13 predefined voice messages such as “vehicle approaching” by measuring the response time and closest approach to the hazard but failed to disclose the on-body placement of the portable tag [93].

Some of the studies presenting technical solutions directed towards safety have mentioned potential integration with hard hat. For instance, one study mentioned the attempt to use visual, auditory, and vibratory alerts along with a black and white screen for the wearer, to be integrated with hard hat [39] while another study mentioned use of flashing lights and auditory alarm-based alerts directed towards hard hat [94]. The exploration of worker body alarm system has also been evaluated for roadside work zones which can be activated based on their relative distance from the intrusion threat [95]. The study noted that the worker body alarms can be worn in the pocket, on a vest or mounted on a hard hat for vibratory and auditory alerts. There has been interest in other on-body placements as well, such as one study that utilized a portable device with audible and vibration alert capacity to be placed in the safety vest near the neck [96].

Prior studies have also made continued attempts for improving the safety of roadside workers by comparing and evaluating the efficacy of commercially available technologies [97,98,99]. Meanwhile, other studies have gauged the feasibility of devices still under research and development [100,101,102]. For instance, one study evaluated the effect of various movements and body orientations of ground workers for its impact on the reliability of alerts [103]. It noted the need to attach the personal protection unit to several locations on the PPE of the ground workers to maintain the effectiveness.

Additional regular efforts continue to be made in preparing a sustainable construction workforce by dissemination of preventive measures, understanding of personnel needs, and promoting research and training [104,105,106,107,108,109,110]. However, no prior study provides a comprehensive investigation regarding the various on-body placements of the wearable devices for promoting construction safety of workers-on-foot. The objective of this paper is to embrace that knowledge void and provide a ground for future studies concerning wearable communication.

## 3. Review Scope and Methodology

The review of prior literature for this study focused on wireless communication which alerts construction workers-on-foot of any potential or impending hazard. The keyword “Construction Safety” was used in conjunction with “Wearable Alerts”, “Auditory Communication”, “Visual Communication”, “Haptic Communication” and “Tactile Communication” to screen publications in the domain through Web of Science, Scopus and Google Scholar.

A closer inspection of the placement of these safety alerts revealed the three aforementioned approaches—placing the safety alerts in the construction environment, placement of the alerts on machines that move around the field or attached to the body of their operators, and safety promotion devices worn by construction personnel moving on foot. As noted in Section 1.1, there is a significantly high number of fatal and non-fatal injuries happening to pedestrian workers in the construction industry. Hence, the authors were interested in understanding the safety alerts to the workers-on-foot; that is, construction personnel working on the jobsite on foot and those not using an equipment or machinery to move around the field.

### 3.1. Review Approach

A careful assessment of literature was carried out. Since this paper is mainly concerned with wireless data communication through wearable devices for workers-on-foot, specific exclusion and inclusion criteria were followed to systematically identify qualifying literature as mentioned in the seven-point checklist below:

I. The research study must be a peer-reviewed paper between 2005 and 2021 and should have been directed towards jobsite safety.
II. The contents of the paper should have included wireless targeted alerts to the worker-on-foot, that is, a jobsite personnel not using a movable machinery to navigate the construction site.
III. Studies with speculative language were left out. For instance, mentions of can be integrated/could be installed on xyz location were ignored.
IV Handheld terminals and construction tool-based alerts were not considered wearable devices.
V. Only studies that displayed prototype placement or provided exclusive text mentions, about where the device was placed, were included.
VI. Commercially available worker safety devices and their evaluation was not in the scope of this investigation.
VII. Follow-ups to the included studies were removed to avoid unnecessary repetition unless a new on-body placement was considered.

With recent advancements in software and hardware capabilities, wearables have made a significant foray into everyday life. The authors of this investigation were interested in documenting the growth and latest trends in wearables as related to construction safety through reliable documents. Hence, the interest in peer-reviewed studies from 2005 onwards and up to the latest completed calendar year 2021, culminated in the first criterion.

Meanwhile, given the harsh work environment of construction jobsite, there are many possible avenues to improve safety. The first approach, as discussed earlier, includes the placement of safety alerts in the construction environment; that is, the alerts conveyed through zone alarms or LEDs and other visual boards placed across the jobsite. A different approach is to directly inform the individuals at-risk or the concerned supervisors. However, conveying the safety alerts and safety status to the supervisors and management using emails, phone messages, and GUI dashboards cannot avert an impending danger in many situations due to the time lost in manual intervention. Therefore, targeted wireless communication to the personnel in the field becomes crucial. Utilizing alerts placed in the construction equipment to directly informed the equipment operators about the safety issues is one approach to avoid pedestrian-vehicle accidents. However, informing the pedestrians or workers-on-foot is expected to be more productive in reducing accidents on construction jobsite given that the pedestrian personnel also face a range of other potential hazards, some of which have been laid out in Figure 1. Hence, the second criterion was adopted to analyze wearable safety promotion devices to convey real-time feedback to the workers-on-foot.

Promoting safety on a construction jobsite for workers-on-foot is a very active area of research and many efforts have been made towards activity monitoring and hazard prediction. However, efficiently communicating the alerts and their on-body placement is sometimes presented as a hypothetical work left out for future studies. Therefore, the third criterion was applied to remove prior studies mentioning potential alert generations and their on-body placements.

The fourth criterion was incorporated to distinguish the wearable design from held design. For instance, the use of handheld terminals can prohibit the personnel from carry out their jobs efficiently, and therefore, it might require an active response from the personnel to pick up or hold the device. Similarly, it is tough to make an argument for the alerts embedded in the construction tools to be included in the wearable design.

Meanwhile, due to the limited inquiry into on-body placement of safety promotion devices, a reasonable possibility is that the peer-reviewed studies could have failed to display the prototype or disclose the exact on-body placement. The fifth criterion was introduced to deal with such scenarios.

The attempt to review prior literature related to on-body placement of safety promotion devices was made to present the current state-of-the-art solutions, and gain insights into the decision-making related to the on-body placement. Therefore, the sixth criterion was implemented to disregard studies related to the technical evaluation of commercially available worker safety devices. Furthermore, the seventh criterion was introduced to exclude efficacy evaluations of previously-included studies as long as no novel contribution was made toward the on-body placement or communication modality.

Two of the authors, working independently, were responsible for the shortlisting the literature that satisfied the seven-point selection criteria. Every selection was then cross-examined by the other author. In instances of occasional disagreements, mutually agreed upon decision, grounded in the thorough analysis of the peer-reviewed study in question, was implemented. Eventually, 29 unique peer-reviewed research papers were recognized from 16 different publication sources. The list of selected papers and their source of publication is provided in Table 1.

This shortlisted literature, satisfying the checklist, was carried out in 16 countries with the United States contributing to ten studies, Spain contributing to six studies, South Korea aiding four studies and researchers from Japan, Malaysia and Mexico collaborating on three studies. Work from Australia, Canada and China was responsible for two studies each while one study each resulted from the work performed in Denmark, France, Germany, Italy, Netherlands, Taiwan and UAE. There were six studies with cross-border collaborations. Among the studies conducted in the United States, three of the research studies were performed in Georgia while North Carolina and Nevada contributed to two studies each.

**Figure 2:**
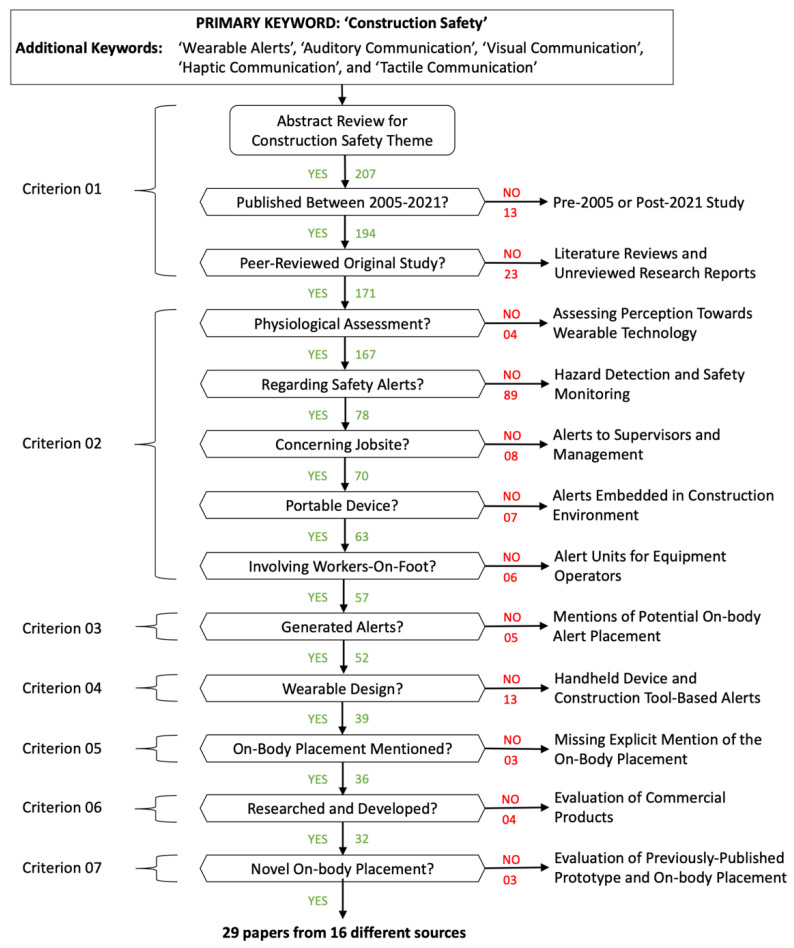
Overview of the review process and selection of literature.

A schematic outline of the literature selection process is depicted in Figure 2. A total of 207 papers were analyzed after reviewing their abstract for construction safety theme. At each decision point, a positive response implied that the concerned paper was retained else it was removed from the selection. The number of papers retained after each decision are mentioned below it while the number of papers removed after each decision are mentioned on the right along with the reason for their removal. These decisions were aligned with the seven-point checklist mentioned earlier.

### 3.2. Novel Communication Network Concept

A novel visualization approach is presented to gain a thorough understanding of the technicalities behind the wireless data communication responsible for alert generation. It has four unique set of nodes corresponding to the four technical aspects for wirelessly communicating to a construction personnel. The four sets are Data Transmission, Data Reception, Data Processing and Alert Placement, in that specific order.

The Data Transmission corresponds to the nodes responsible for the initial wireless data transmission such as using radio or magnetic frequency waves. Other transmission modes of research interest are testing new proofs of concept such as wearers’ reaction time. This data could be wirelessly emitted from a nearby workstation using Wi-Fi or Bluetooth technology. Another possibility is that the selected study could be generating alerts based on the assessment of the present condition such as voluntary physical behavior or involuntary physiological condition of the construction personnel without making any attempts towards localization or positioning.

The wireless Data Transmission is then received by a set of Data Reception nodes. This could be received by an appropriate device positioned on the equipment, worn by the construction personnel, or by active readers placed on the jobsite. Hence, by definition, the node where data are received has to be different from the node that is transmitting the data. The Data Processing refers to the set of nodes where the decision to initiate or reject further communication is being made.

If the assessment recognizes an impending hazard, corresponding feedback will be initiated. Assuming that a decision to generate a warning or alert has been made at the Data Processing node, the subsequent communication takes place using one or any possible combination of the three human senses—hearing, sight and touch. The on-body placement of communication device corresponding to those three senses forms the next set of nodes for Alert Placement. In order to limit the number of different nodes, especially during the Data Transmission, Data Reception and Data Processing phases, some simplifications were carried out as presented in Table 2.

**Figure 3:**
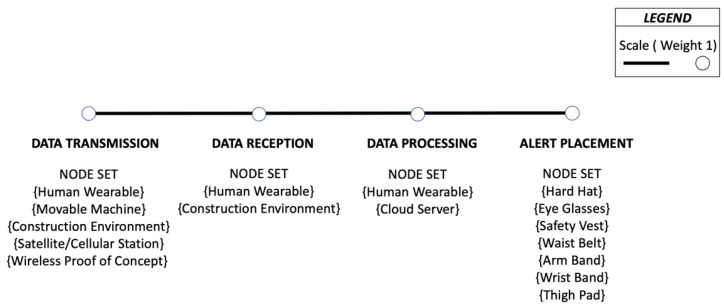
An account of the nodes involved in the novel wireless communication network concept.

Additionally, an image is provided in Figure 3 to illustrate the various nodes involved in the safety promotion and the schematic of a typical route between the aforementioned nodes is also represented in Figure 3. The frequency of a node will impact its size whereas the frequency of a route will alter its line thickness. All the node sets and routes represented in the figure have equal weight (1).

The data transmission and data reception cannot happen at the same type of node unless a transceiver is employed. Otherwise, there is an exhaustive number of routes possible between consecutive node sets. The node set for Alert Placement was determined from the 29 shortlisted papers as detailed in the next section.

## 4. Results and Discussions on Wearable Safety Promotion Devices

The wearable devices were distributed across seven unique on-body placements for alert communication directed towards the three human senses—hearing, vision and touch. A majority of the reviewed cases only tested a single on-body placement of the wearable communication device while two study mentioned alternative on-body placements as well [121,129]. The primary on-body placement for each study is indicated in Table 3. The research studies that conveyed information beyond a binary presence or absence of danger are also recognized with a star mark in the Table 3. This includes efforts to communicate about the proximity to the hazard along with the direction and details of the approaching equipment among other information. Fifteen such undertakings were made to convey rich wearable safety alerts across six on-body placements.

Various combinations of the communication modalities were explored with two instances where all three senses were evaluated on a single on-body placement [122,126]. There were four instances of auditory-visual alert combination [118,128,132,139], and one instance of visual-tactile feedback combination [127]. Standalone auditory alerts were implemented by five studies [111,112,131,136,137], standalone visual communication was applied in another five studies [116,119,130,133,134], while standalone tactile feedback was mentioned in four studies [114,120,123,135]. Meanwhile, eight studies integrated auditory and tactile alerts to communicate safety information to the personnel [113,115,117,121,124,125,129,138]. Given that the construction site is a visually challenging work environment, it is understandable and consistent with research in other fields researching about visual limitations [140].

Regarding the choice of alert combination for auditory, visual and tactile communication modalities, the researchers were confronted with three options for each mode—no alert, static alert, and rich alert. Therefore, for any given on-body placement, a total of 26 combinations are possible, that is, $3^{3}-1$ where the one exception relates to the instance where all three modes are ‘no alert’.

What made these 29 studies select a particular on-body placement and a specific combination of communication modality? Their technical viewpoints, information conveyed, and limitations are evaluated according to each communication mode to gain better insights on the state-of-the-art solutions.

### 4.1. Safety Promotion Using Wearable Auditory Communication

While it is evident that the auditory noises on construction sites can reach unsafe levels due to the nature of the work and equipment involved, auditory communication has still been the preferred mode of communicating alerts to the construction personnel. It was the most widely explored method, utilized in 19 out of 29 studies, for targeted alert delivery to the workers-on-foot.

#### 4.1.1. Technical Details of the Wireless Data Communication

**Figure 4:**
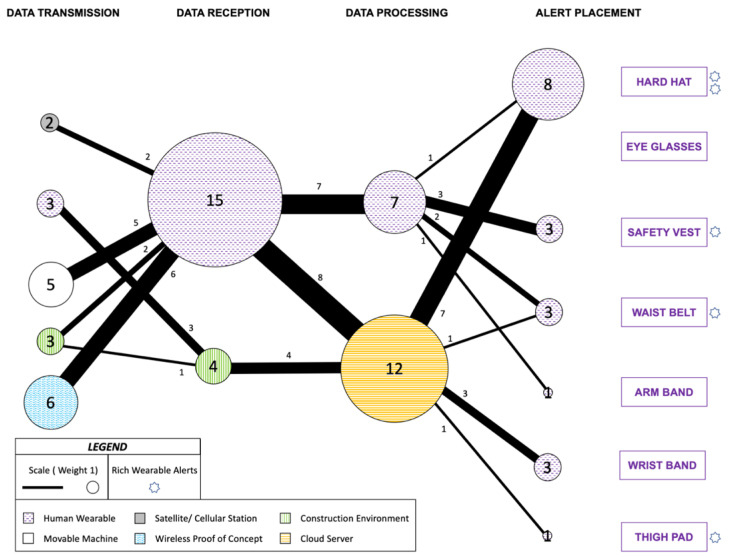
Auditory Communication Network displaying a wide range of solutions attempted in the prior literature. Digits inside the nodes and next to the routes display the frequency of use.

The complete communication network for the 19 studies is mapped through different nodes and routes as presented in Figure 4. Additionally, the studies that conveyed rich wearable alerts, meaning information beyond mere presence or absence of safety was conveyed to the workers-on-foot, are recognized with a star mark to the right of the Alert Placement node. Since this study is concerned with alerts to the workers-on-foot, when some of the reviewed literature alerted equipment operators or sounded an alarm in the construction environment, their respective nodes and routes were ignored for the purpose of this network. Only the nodes and routes responsible for providing feedback to the workers-on-foot are included. The digits inside the nodes indicate the number of reviewed papers that utilized that specific node, and the digits next to the routes denote the count of reviewed papers utilizing that particular route to communicate the alert.

Wireless Proof of Concept was the most frequent node for Data Transmission set (6) while Human Wearable node was most frequently used for Data Reception (15). Cloud Server node (12) is where most of the Data Processing happened, while hard hat (8) was the most preferred Alert Placement followed by safety vest, waist belt and wrist band (3). The most frequent route for the data transmitted was Wireless Proof of Concept to Human Wearable node with six instances. Between Data Reception and Data Processing node sets, Human Wearable node to central Cloud Server node was utilized in eight instances. Cloud Server to hard hat was the most common route between Data Processing and Alert Placement, as applied by seven reviewed studies.

13 out of the 19 auditory alerts studies also made efforts towards localization and real-time positioning through a diverse range of techniques. A majority of the data origination was at the Human Wearable itself or at the Movable Machine representing equipment on construction site. This was mainly carried out using radio frequency communication in seven instances [111,115,117,124,125,131,136,137], where one study also involved GPS [111].

Three studies utilized ultra-wideband (UWB) technology, for automating safety control logics for overhead hazards [112], for determining proximity from construction equipment and construction environment hazards [121], and for indoor positioning on a local coordinate system [137]. There was one instance of using Bluetooth technology [138], one instance of using magnetic field-based communication [129] and one instance of ultrasonic sensor use [139] for determining proximity from construction equipment. Six studies, classified under the Proof of Concept node, did not attempt localization feasibility and instead transmitted data from smartphone’s built in accelerometer [113], a workstation [126] and sensors embedded in the wearable alert device [118,122,128,132].

#### 4.1.2. Information Conveyed

The auditory communication alerts, with various strategic on-body placements, mostly attempted to convey the proximity of a heavy equipment. One study used handheld GPS-based worker-equipment location to establish a bidirectional voice communication using earphones and microphones located on the hard hat through creation of static and dynamic prohibited zones [111]. Another study warned workers about the proximity to construction equipment through a magnetic alert tag which was worn on the belt pouch by some users while others had it in a safety vest pocket [129]. This personal alarm device (PAD) produced an alarm that changed pulse based on the distance from the generator and a vibrating tactile alarm was activated in the closest zone generating a continuous vibrating alert.

Radio frequency has also been used to inform workers about the equipment proximity through a personal protective unit worn on one or both the arms [117]. Meanwhile, attempts have also been made to utilize passive ultra-high frequency (UHF) wearables such as the development of SmartHat for construction equipment proximity warning [131]. It noted the need to place the auditory alarm closer to the ears by placing a microprocessor and buzzer on the hard hat. Similarly, another study incorporated a hard hat attached tag that can assess proximity to equipment using a Chirp Spread Spectrum-based radio frequency location system [137]. Meanwhile, radio frequency-based system has also been used to prevent equipment backing up accidents through auditory alarm in a wrist band [115].

Another study attempted to prevent worker-equipment collisions by placing Bluetooth beacons [138]. Audible alerts were conveyed to the workers through an Ipod that could be worn around the belt but was worn in the right pocket for all trials. It has been included in this investigation as part of the thigh pad Alert Placement node. The distance between the worker and the equipment was trisected at 4 m, 8 m and 12 m with audible beeps intensifying with a reduction in the distance. Through a wearable wrist watch, the use of auditory alert was applied to improve road work safety as well [139]. A collision prevention system was also developed with UWB channels used to activate buzzer placed on the hard hat [121]. One study focused on determining the wearers reaction time for auditory alert system placed on the safety vest [126].

However, several recent studies have attempted to convey other information as well. For instance, one attempt was made to detect fall hazards using smartphone-based accelerometers mounted on the waist belt [113]. An attempt to determine whether workers are wearing protective equipment in hazardous zones was made by using silicone based single point pressure sensors in hard hat, shoes and gloves and the location coordinates. The absence of protective gear triggered an auditory alarm on the hard hat tag [124]. In another study, RFID tags and readers were utilized to monitor the use of PPE, the absence of which triggered a buzzer embedded in the safety vest [136].

Meanwhile, one study utilized the chirp spread spectrum (CSS) to send auditory alerts to a worker’s hard hat when it was determined to be inside two stationary or one moving danger zones through relative positioning in 3D space with help of tags and anchors [125]. Another study utilized radio frequency to alert about danger and warning zones [112]. If inside a hazardous zone, a red alarm was sent, and if inside the surrounding boundary then the movement was further measured, and warning alarm was sent upon approach to the boundary. Jordan Curve Theorem was used for 2D polygons to determine the alert and warning criteria, and efforts were made towards improving the reliability of the localization accuracy.

The utilization of wearable sensors to detect and inform about anomalies in physical parameters such as body temperature [128], heart rate [122], and environmental conditions such as moisture, barometric pressure, surrounding temperature, presence of harmful gases [132] is also quickly becoming an active area of study.

#### 4.1.3. Limitations of Wearable Auditory Communication

While auditory alerts have been part of the heavy equipment for quite some time [71], the construction sites injuries continue to be at undesirably high levels. Recent technological advancements have prompted the researchers to place the auditory alarms on the personnel. However, if the background noise reaching the construction workers’ ears is high, that means the auditory communication to convey any imminent danger should be even higher.

Research suggests that such alarm should be about 10 decibels higher than the noise they are trying to alert through [8]. Given the high average noise levels on construction site, generating an auditory alarm might be detrimental to the hearing sense of the personnel. Conversely, if the work area mandates the use of hearing protection and the personnel is in compliance then the ability to convey such auditory alerts also decreases along with the decrease in the background noise. Auditory communication (19) was the most widely explored mode but there were only five studies that used auditory communication without combining it with visual or tactile alerts.

These limitations are likely what prompted many researchers to explore it in combinations with other modes that utilized sight and touch senses. This was explicitly mentioned in a study which recognized that when wearing earplugs, workers had a hard time hearing tag alarms, placed at the bottom of belt pouch, due to the equipment noise and backup alarms [129]. A separate study mentioned that a vibrating alarm had to be integrated so that the worker can be notified even if wearing headphones or working in an area with loud construction noise [117]. Another study also noted that the on-body placement of the device at waist level reduced the audibility of the alarm [131].

The use of rich wearable auditory alerts was also limited to five studies [111,112,128,129,138]. One study had a bidirectional voice system to allow uninterrupted conversations [111]. Another study utilized two distinct auditory sound to convey an above or below average body temperature [128]. The other three studies altered the intensity of auditory alarm to convey the distance from the hazard where higher intensity implied a nearby danger. However, no additional information was being provided about the type of equipment or potential danger.

### 4.2. Safety Promotion Using Wearable Visual Communication

The use of visual communication for wearable safety promotion has been quite limited, possibly due to the heavy visual workload nature of working on a construction jobsite. On the whole, the sense of sight was utilized by 12 out of the aforementioned 29 studies to alert the workers-on-foot. Unlike the auditory communication, one significant advantage for visual communication is the ability to display textual as well as graphical formats which can be a tremendous boost to compensate for the language incoherence in the construction industry.

#### 4.2.1. Technical Details of the Wireless Data Communication

**Figure 5:**
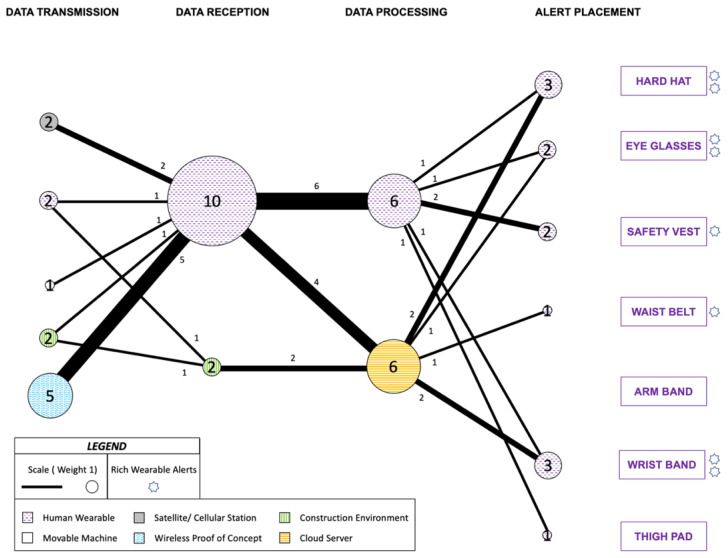
The Visual Communication Network for conveying textual or graphical information to construction worker-on-foot is displayed based on the insights from prior literature. Digits inside the nodes and next to the routes display the frequency of use.

Overall, a good range of nodes have been utilized so far, but the network seems sparse compared to the use of the other two senses, as displayed in Figure 5. This visual communication network for warning construction workers-on-foot is on the same scale and annotations as Figure 4 to allow the readers to compare the differences between the communication network diagrams presented in this paper. Additionally, the nodes and routes responsible for any simultaneous alerts to equipment operators or construction environment were not included. Nearly half of the studies (five out of the 12) exclusively used visual communication [116,119,130,133,134].

Two studies utilized the GPS technology, with one case of integration with UWB [119], and one instance of integration with inertial measurement unit (IMU) [134] for outdoor positioning on global coordinate system. In two other instances, ultrasonic sensors were exploited for determining proximity with construction equipment [139] and construction environmental hazards [127]. Meanwhile, Bluetooth technology was also utilized for localization purposes where one study used it for PPE detection [130] and another study utilized it for determining proximity to construction equipment and construction environmental hazards [133]. Vision-based technology was also used for determining proximity to construction equipment and construction environment hazards [116]. Five studies did not attempt localization. One of them transmitted data from a workstation [126] while the other studies used measurements from sensors embedded in the wearable alert device [118,122,128,132].

The Wireless Proof of Concept node was the most frequent node for Data Transmission (5). The Human Wearable node was the most frequent node for Data Reception (10) while the central Cloud Server and the Human Wearable node were equally utilized for Data Processing (6). Hard hat and wrist band (3) emerged as the most favored nodes for Alert Placement along with the eye glasses and safety vest (2). The most frequent route for the data transmitted was from Wireless Proof of Concept to Human Wearable with five instances. Between Data Reception and Data Processing node sets, Human Wearable to Human Wearable was the most frequent route with six instances. Human Wearable to safety vest, central Cloud Server to hard hat as well as central Cloud Server to wrist band were the predominant route between Data Processing and Alert Placement with two instances each.

#### 4.2.2. Information Conveyed

Most of the visual information communicated to the workers-on-foot, similar to the auditory communication, was focused towards identifying and predicting risks related to the proximity of heavy equipment [116,117,119,133,134]. A majority of these studies made use of warning lights, usually light emitting diodes (LED), positioned on the PPE including one instance with varying level of illumination [126]. Meanwhile, one of these studies attempted to convey the proximity of equipment using GPS technology by placing an LED indicator on the hard hat [119]. It is noteworthy because of the use of IMU, also mounted on the hard hat, to predict the gaze of the wearer. The visual alarm was deactivated after 10 s if the gaze of the wearer was determined to be onto the hazard. It did not issue any alert if the wearer was determined to be already looking in the direction of the hazard.

Two studies applied rich wearable alerts on hard hat. One study placed eight LEDs shining into the visor, for visual cues and information [127]. The study tested 15 different patterns such as directions and color-coded notifications including animated patterns with pixels moving to one direction. Proximity warning was indicated with red colors in the center of the visor for forward obstacles while back obstacles were indicated by red colors on both ends of the visor. The other study also deployed a LED strip to notify workers of anomalies in environmental factors through color codes [118]. However, the LEDs were placed on the outer shell of the hard hat. Another study with similar scope emitted visual color code through LED strip, integrated on the waist belt, to notify of low battery status.

Contributions to reduction of false and negative alarm rate was made for stuck-by equipment hazard through GPS aided Inertial Navigation System (INS-GPS) [134]. The study utilized the LED on raspberry pi, placed at the elevation of the knee or slightly higher, to convey alerts. It is counted in the thigh pad node for Alert Placement. Meanwhile, a different study evaluating the wearer’s body temperature for early detection of heat stroke placed the LEDs on the back side of the safety vest [128]. Predefined thresholds were incorporated to convey normal temperature in green, too cold in blue, and too hot in red.

The employment of liquid crystal display (LCD) screen has also seen significant research interest. It has been primarily associated with wrist placement. In one study, the adequate use of PPE was monitored using Bluetooth beacons attached to the wearable personal equipment as well as to the workstations [130]. Corresponding notifications are displayed through a standalone application for a wrist watch with texts and graphics notifying the worker about the detected and missing personal equipment needed for that specific workstation. Another study applied LCD screen on a wrist watch to improve road work safety and alert about oncoming vehicles [139]. A combination of LCD screen and LED strip was also noted in one study detecting anomalies in environmental and bodily parameters [122].

The use of wearable augmented reality glasses through the Glass Development Kit (GDK) in Android Studio was also explored [116]. The perspective of the video feed obtained from the glasses was compared to the stationary and close-circuit cameras to identify the workers. Visual graphical alerts were projected onto the smart glasses to notify of any potential hazards through the application of fuzzy interference. Meanwhile, a different study incorporated Bluetooth beacons on heavy equipment and measured their signal strength at the smart augmented glasses to determine proximity [133]. This was utilized to convey graphical alerts about the distance and type of vehicle near the worker who is expected to be conducting maintenance and safety checks on foot.

#### 4.2.3. Limitations of Wearable Visual Communication

The major concern with conveying any information using sight is the ability to establish the initial contact. This might be the primary reason why some studies have attempted a combination of auditory and visual alerts. In addition, while IMUs might be decent indicators of body posture, retrieving gaze information could be more complicated and could certainly utilize additional sensors monitoring the brain waves for enhanced determination as noted. The placement of the visual information certainly plays a major role in attracting the attention of the user, and that is precisely the reason why one study acknowledged that the use of visual alert on the hard hat was purely for demonstration purposes, and suggested replacing it with an auditory buzzer [119].

Other studies placed color-coded LEDs such that they are intended to alert the nearby personnel rather than the wearer about any potential danger [118,128]. One study that utilized LED placed above the knee (thigh pad) recognized the need to explore more reliable actuation such as audible alarms in the next stages [134]. On the other hand, the use of LCD screens provides the benefit of textual as well as graphical notifications and alerts. However, one of the prior studies that utilized LCD screen on a wrist watch received concerning feedback from industry experts [130]. The ability of the prototype to withstand harsh industry conditions was noted, and suggestions were made to use thin elastic wrist band with LED notifications instead. Other feedback included exploring solutions such as ear pieces and displays.

Though head-mounted displays and smart glasses have a lot of potential, they can run into limitations if wearing glasses significantly impacts a particular trade where complete visual awareness might be anticipated on the tasks being carried out. In addition, the personnel already wearing regular glasses or sunglasses due to high luminance might not prefer augmented glasses without significant adjustments. Potential development of smart contact lenses might be able to eradicate some of these deficits in the future.

### 4.3. Safety Promotion Using Wearable Tactile Communication

While the auditory and the visual communication modes have been available for a while, wearable tactile communication is a recent attempt to convey meaningful information through the sense of touch. Some of the earlier studies reviewed here did include vibration to convey some form of predicted danger but the amount of information that could be conveyed was very limited. Recently, newer avenues have been made possible due to significant strides in hardware and software capabilities. This has resulted in the adoption of the tactile communication mode for navigation and communication by the defense and civil research communities. At times the objective is to overcome high auditory hinderance in the environment, while in some other cases the objective is to deliver targeted information without creating any unintended noise.

Case studies of wearable tactile communication for navigation of dismounted soldiers has also been explored. One study presented a waist belt worn over underclothing with eight tractors for 360-degree navigation through a personal tactile navigator (PTN) [141]. A subsequent study compared the GPS based tactile alert navigation performance to handheld GPS device and head mounted map-based GPS device [142]. In another study, tactile communication was utilized to navigate motorbike riders that could not look at their smartphones for directions while steering the powered vehicle [143]. The tactile motors were placed near the shoulders on a jacket. The alert activation and intensity, on the left and right shoulder, varied depending on the navigation information to be communicated subject to the distance from the upcoming turn or exit direction for the rider.

For construction related efforts, among the 29 shortlisted studies, 15 utilized wearable tactile communication for workers-on-foot. However, a closer look reveals that the use of tactile feedback is on the upward trend. For instance, 8 out of the 13 latest studies (2017–2021) included tactile communication for wearable safety promotion. While many of the studies reviewed here continue to use simple vibrations to warn construction workers-on-foot, some early research is being carried out to convey more diverse information or to understand the motor configuration and response time associated with this technology and were also included in this review.

#### 4.3.1. Technical Details of the Wireless Data Communication

**Figure 6:**
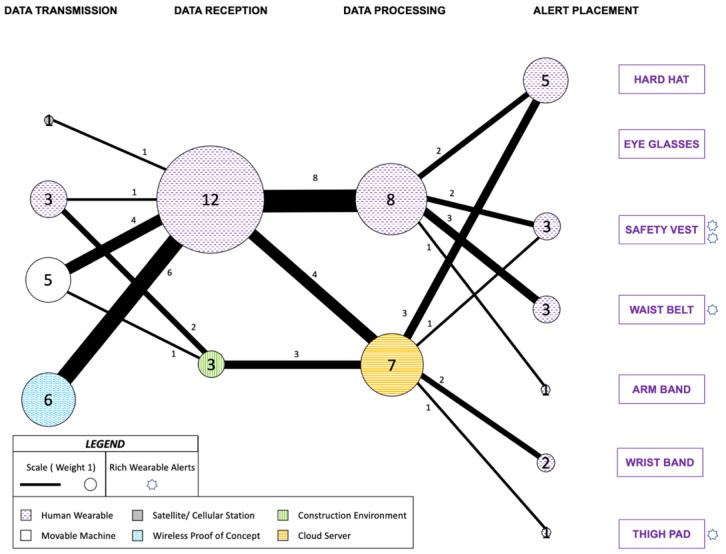
The Tactile Communication Network for conveying information through sense of touch, replicated using vibration motors, to the construction worker-on-foot as per the reviewed literature. Digits inside the nodes and next to the routes display the frequency of use.

The tactile communication network for wireless feedback is displayed in Figure 6 and follows the same legend and scale used for Figure 4 and Figure 5. There were six instances where the data were transmitted from a Wireless Proof of Concept node while the Movable Machine node was also highly employed in the Data Transmission set (5). Human Wearable was the most frequent node for Data Reception set (12), with six instances of data arriving from the Wireless Proof of Concept and another four instances from the Movable Machine. For 8 out of the 12 times the data were received by the Human Wearable, it was processed at the same node thereby also making it the preferred route (8) between Data Reception and Data Processing.

The Human Wearable to safety vest and waist belt along with the central Cloud Server to hard hat were the most frequent route for Data Processing to Alert Placement with three instances each. Hard hat accounted for the most on-body alert placements with five instances while waist belt and safety vest were the second most preferred placements with three instances each.

One investigation explored the placement of vibration motors on the front of the chest as well as close to the neck around the collar bone on the safety vest [126]. This study was concerned with the response time of various warning communication modes. It used a workstation to wirelessly communicate to the wearable microcontroller, through a Bluetooth module, which is noted under the Wireless Proof of Concept for Data Transmission purposes since it did not attempt localization. Similar efforts were carried out by others as well. The vibration motors were placed on the safety vest and on the waist belt while the data was transmitted wirelessly with the help of WiFi capabilities [120,123]. Another study did not include localization and instead determined tactile alerts based on the data from smartphones’ built-in accelerometer [113]. Similarly, in another instance alerts were generated based on the data from IMU and EEG sensors incorporated in the hard hat [135] and did not make localization efforts.

The rest of the nine studies determined the alerts based on localization. Such data, achieved through radio frequency technology, was used for determining indoor positioning [124,125], and determining proximity to construction equipment [115,117]. Magnetic field-based technology was also utilized for determining proximity to construction equipment in one instance [129], while such efforts have also been realized using Bluetooth technology [138] and ultrasound sensing [127] as well. Meanwhile, localization using satellite-based global coordinate system, for determining proximity to construction equipment and construction environment hazards, has also been explored on integration with radio frequency [114] and UWB technology [121]. One study is noteworthy for including photovoltaic cells and a passive sleep mode, until the wearable wrist band is activated in the proximity of radio frequency emitted work zone, to enhance the battery life [115].

#### 4.3.2. Information Conveyed

As previously stated, tactile communication is a relatively new mode of conveying targeted information to the workers-on-foot. Many of the studies reviewed here only predicted an impending hazard based on the presence of vibration while the absence indicated safety [113,115,117,121,122,127,129,135].

Discussions from one study reported that some workers who wore the vibration tag around the belt pouch did not adequately feel it while those who wore it around the vest pocket reported the vibration could be felt better depending on how close they wore the tag to their body [129]. In another instance, a virtual construction system received the localization information and determined the relative position in 3D space to send vibration alerts to tags installed on helmets [125]. Meanwhile, a different study utilized building information modeling to determine whether personnel is wearing adequate PPE through various pressure sensors, to trigger alert through tags installed on hard hat in case of an absence assessment [124]. The use of handheld clickers to measure the response time of safety vest-based vibrating alerts during three simple tasks, and across the three communication modalities, has also been evaluated [126].

One study investigated three signal parameters—active signal length, signal intensity and signal delay—to understand the distinguishability between them [120]. This was used to test whether simple information can be conveyed through tactile feedback on a safety vest. The technology used in this study was very similar to another study that added a second wearable board to acts as a client, and placed the vibration motors on a 8.5 inch waist belt [123]. The contributions towards determining the adequate number of vibration motors and their alignment are especially noteworthy. It was recently tested in a controlled environment for assessing the system’s reliability [144].

Attempts has also been made towards the use of sensor network for autonomous close-call data generation, reporting and evaluation. This was evident in a study that configured the alarm zones into four distinct categories based on the proximity to the hazard [114]. The four configurations were no alarm zone, warning zone, slow zone, and stop zone. The tactile feedback to the wearer was placed in the safety vest near the neck region.

#### 4.3.3. Limitations of Wearable Tactile Communication

Since tactile communication is still at a nascent stage, the meaningful information that can be conveyed is still being explored. The success of efficient tactile communication is contingent upon how well we can feel the vibration on our skin and make the association and interpretation regarding a particular tactile feedback. As a result, only four studies attempted to provide rich wearable alerts [114,120,123,138].

The perception of the vibration itself is affected by at least two parameters. First, the magnitude of the vibration of the tactile device, controlled by the count of vibration motors and their associated waveforms, which affects our perception. Second, the on-body placement of the tactile feedback device also affects our perception. The same magnitude of vibration can be felt differently at different on-body placement or not felt at all in certain placements as noted by several of the reviewed studies. For instance, vibration was felt better in certain safety vest position compared to the belt pouch [129]. Several studies also noted that if the safety vest is worn over thick clothing, the touch might not be perceived by the wearers’ body.

At the present stage, most of the prior studies focused on the presence or absence of vibration to convey information. Hence, with a lack of uniform framework and consensus regarding the placement of tactile device, the information that can be transmitted is severely limited. In addition, with the technology being in an early phase, we could not find any studies regarding the inputs on tactile communication from industry professionals regarding its placement and (or) usability.

### 4.4. Takeaways and Future Recommendations

While many significant efforts have been made so far, as discussed in this paper, the fatal and non-fatal injuries on construction sites are still too high. Additionally, the review of wearable safety promotion devices for workers-on-foot has revealed the capability offered by the ongoing research, and the lack of consensus in the academia regarding the favorable on-body placement for wearable safety promotion devices.

#### 4.4.1. Study Outcomes

With the recent technological advances, the construction workers-on-foot can receive information about potential or impending danger beyond the bodily auditory, visual and physical communication limits. As determined from the literature review, seven unique on-body placements for safety promotion devices have been utilized so far. Some of them, such as the hard hat, have been strongly favored over certain on-body placements such as the arm band. Despite these signs, it is tough to argue that a consensus exist as to what is the optimal on-body placement for a safety promotion device. It is a question that remains to be answered.

As seen from the communication network diagrams, many of the reviewed studies made efforts towards positioning and localization to determine alert generation. Radio-frequency based positioning was the most popular technology to provide informing about such location-based impending hazards. However, a significant number of studies also focused on generating alerts based on sensing anomalies in the wearer’s physiological and environmental parameters.

The density of solutions noted in the auditory communication network reemphasizes the role of auditory noise in jobsite accident, and the efforts to alleviate them by placing buzzers and speakers in the hard hat including two rich wearable alerts. For tactile communication, it is noteworthy that despite the hard hat being the most used on-body placement, none of the reviewed studies utilized it for providing rich wearable alerts. Meanwhile, two rich wearable alert efforts were made for the safety vest and one each for the waist belt and thigh pad. The underlying reasoning for such decisions needs to be ascertained in future studies.

While all the auditory and tactile communication efforts were directed towards alerting the workers-on-foot, a divergence was observed in some visual communication efforts. In two instances the solution was guided towards the co-workers-on-foot, that is the coworkers of the workers-on-foot. This was done by placing the LEDs on the outer casing of the hard hat, and on the back of a safety vest. This possibility arises because such LEDs can be seen by nearby coworkers as opposed to the auditory or tactile communication which are limited by the background noise and require direct physical contact. However, a majority of the visual communication efforts were still targeted towards the workers-on-foot. Another prominent difference from the auditory or tactile communication was the transmission of safety alerts through smart glasses. Two rich wearable alert efforts were noticed on the hard hat, eye glasses, and wrist band while safety vest and waist belt had one instance each.

#### 4.4.2. Suggestions for Future Research and Development

There is much scope to further explore the on-body placement of wearable safety promotion devices. For the purposes of this review, seven unique on-body placements were identified. A further in-depth cataloging should be explored in future research. For instance, one study tested visual alert on the hard hat by placing a LED light on the outer surface, another tested color-coded lights on the hard hat brim which could be an effective way of gaining user’s attention in hazardous situations while other studies placed the device around the inner harness. Similarly, in case of safety vest, initial classification could be the placement of the safety promotion device on the front side, back side, or near the neck region. The resulting set would then correspond to 11 unique on-body placements.

Meanwhile, additional five on-body placements that could be explored include ear pieces, neck band, knee pads, ankle bands or shoe soles. They should be considered viable as long as they do not interface with the safety or the responsibilities associated with the trade. Hence, at the minimum, a total of 16 unique on-body placements are available to the researchers interested in warning the construction workers-on-foot about potential and impending dangers.

Regarding the communication modalities, as discussed earlier, there are 26 possible combinations for each on-body placement. A complete lack of auditory and tactile communication is noted on eye glasses, and a lack of visual communication on the arm band is also evident. Additionally, the potential associated with rich wearable alerts has seen very limited research interest so far. The sheer amount of permutations of the sensory magnitude, associated waveform and configuration can deeply impact the amount of meaningful information that can be conveyed.

**Figure 7:**
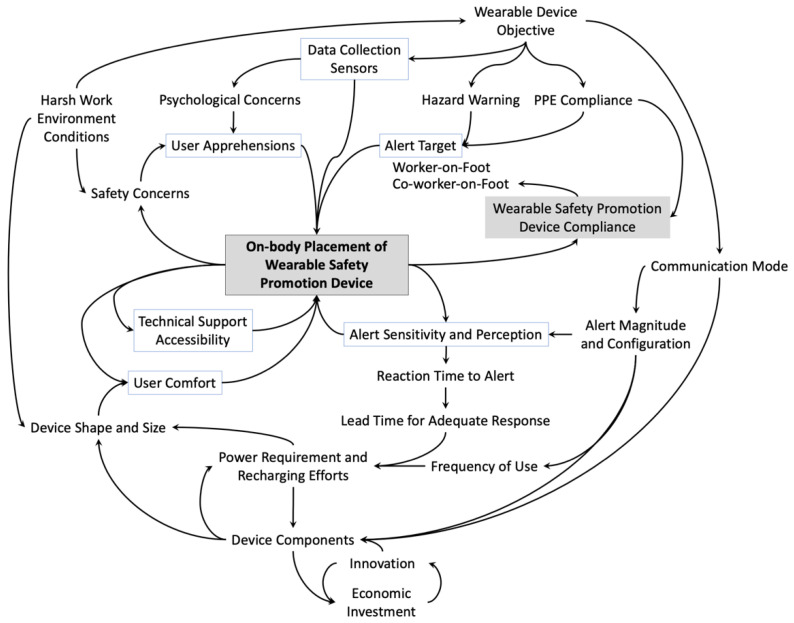
The prominence of on-body placement as depicted through the ‘on-body placement loop diagram for wearable safety promotion device’.

In order to assist the selection of on-body placement of wearable safety promotion devices, a loop diagram is presented in Figure 7 to emphasize on its vital role. Several leading aspects need to be deliberated when shortlisting an on-body placement as they can impacts the selection as well as gets impacted by the selection. Six factors appear to have a substantial role in deciding the on-body placement of the safety promotion device. Three of these factors tend to be more non-technical or consumer-oriented as compared to the other three which are more technical in nature.

User comfort, technical support accessibility and user apprehensions are the three consumer-oriented factor that can dictate the acceptability of wearable safety promotion device and impact its on-body placement. Given the intent to capture the attention of the user, such a device can have negative connotations given the harsh nature of construction jobsites and could end up increasing the safety concerns. Additionally, the objective of such data collection can cause psychological concerns such as privacy issues. Safety concerns and psychological concerns comprise the matters related to user apprehensions. The on-body placement can have considerable impact on the user comfort as well as the accessibility of the device for any maintenance needed during the use. Therefore, they ought to be contemplated during the early design and development.

The three relatively more technical factors are alert sensitivity and perception, intended alert target and data collection sensors utilized. The sensitivity across the human body varies widely and the same magnitude of alert that can be perceived on the fingertips might not be perceived on the legs. Hence, the alert sensitivity and perception are dependent on the on-body placement. Opting for a more sensitive region for the selected communication modalities can be a beneficial factor. The intended target of the alert, between the worker-on-foot or co-worker-on-foot, can also alter the on-body placement preference. For instance, a worker-on-foot cannot be anticipated to respond to the visual communication device placed on the back, but the co-worker-on-foot can if a line of sight is established. Regarding third technical factor, if the alert is to be generated based on the physiological condition of the wearer, there might be limitations regarding where the concerned data collection sensors such as IMU or pulse oximetry sensor could be placed.

Some of the prior research has also successfully attempted to alert about the compliance of PPE. Assuming that, in the near future, such wearable safety promotion device will become a part of the regular personal wear, it will be crucial to detect the compliance of this additional equipment. It has been the primary reason many current solutions for a safety promotion device were integrated into the PPE that is utilized on the jobsites at present. In instances where such safety promotion device integrated PPE is found missing, potentially through vision-based monitoring by jobsite cameras, an alert can be targeted to the co-worker-on-foot. However, such efforts would be not be very practical if the wearable safety promotion device cannot be easily detected such as when a safety promotion device incorporated waist belt is worn under a safety vest. Hence, it is another important factor to consider while moving forward.

In the interim, technological advancements should continue to be incorporated and utilized for better prediction and communication of safety status [145,146,147]. The allocation of resources in construction safety also needs to be closely evaluated as the technology evolves, given the evidence that an optimal investment could in turn decease the direct and indirect costs associated with jobsite accidents [148].

#### 4.4.3. Developing an Evaluation Framework

Of the 29 studies reviewed in this paper, only one study evaluated various combinations of the communication modalities and the wearer’s reaction time for one on-body placement. Furthermore, no attempted were made to compare multiple on-body placements against each other. This reveals a knowledge gap and a lack of framework to evaluate different on-body placements and communication modalities.

As noted in the previous section, a total of 16 unique on-body placements are available to the researchers interested in warning the construction workers-on-foot about potential and impending dangers. Eleven of these on-body placements have been tested individually, and they were simplified into seven on-body placements for the purpose of this review paper. If a study intends to compare multiple on-body placements, there are $2^{n}-1$ combinations available for n unique on-body placements. Furthermore, if the permutations of *c* communication modalities (auditory, visual, and tactile) and a alert type (no alert, static alert, and rich alert) are taken into consideration for each unique on-body placement, the theoretical solution set would increase to $a^{\left(c\times n\right)}-1$ for n unique on-body placements. Therefore, for the discussed parameters (a=3;c=3;n=16), a total of $7.97\times 10^{22}$ combinations are possible. It would be infeasible, if not impossible, to evaluate all of them before recommending an ideal on-body placement for the safety promotion device.

**Figure 8:**
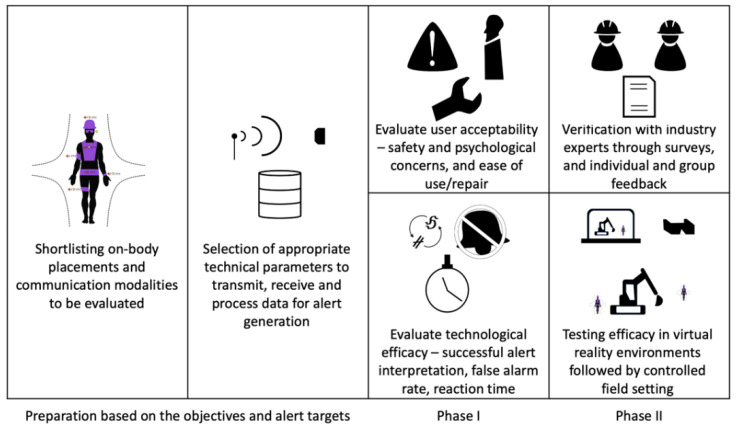
A preliminary two-phase framework to evaluate various on-body placements and communication modalities for wearable safety promotion devices.

Given the limited research in the analyzing the on-body placement of safety promotion device, there is a complete lack of framework for meaningful comparison. An attempt has been made to provide a preliminary overview as illustrated in Figure 8.

Referring to Figure 8 again, the preparation for such an evaluation would encompass shortlisting the on-body placements to be tested, along with the communication modality and alert type, preferably rich alert. The on-body placement loop diagram for wearable safety promotion device, provided in Figure 7, would be a good starting point while making an initial selection. In addition, after one or multiple communication modalities has been chosen, the respective communication network diagrams provided in this paper can assist with selecting the prevalent technical architecture required for the evaluation. The framework for evaluation process itself is divided into two phases, and each phase is further categorized for the user acceptability, and for the technological efficacy. 

The human perspectives involved in the determination of jobsite safety needs to be accounted and timely deliberated to accelerate the path to consensus on wearables [149,150]. During the user acceptability evaluation in Phase I, the researchers are recommended to focus on the three consumer-oriented factors—user apprehensions involving safety and psychological concerns, user comfort, and the technical accessibility of the device for any maintenance requirements. Future studies should develop methodologies to gauge these parameters, and for further verification with industry health and safety experts during the Phase II. The feedback received from the industry professionals through email and telephone surveys as well as through individual and group interviews can help enrich the on-body placement loop diagram for wearable safety promotion device, and decode the significance associated with each factor influencing the on-body placement of wearable safety promotion device.

The evaluation for technological efficacy has already seen some advancements, including the papers reviewed in this study, as related to the alert interpretation in case of rich wearable alerts, false alarm rates, and wearer’s reaction time. However, additional Phase I evaluation studies that directly compares multiple on-body placements need to be initiated in controlled conditions, similar to the efforts in determining wearable sensors for analyzing posture [151]. 

Additionally, based on the feedback from industry experts through user acceptability evaluation, it is likely that some delicate issues will have to be evaluated in virtual reality environments in Phase II before they can be tested on the jobsite. For instance, would any alert compromise the ability of a worker to perform their daily tasks or negatively impact the way a specific tool is used, given the attention-seeking nature of wearable safety promotion devices? In an already harsh work environment, further increasing the risk of injury is not something any research would intend to do. However, ensuring that requires a detailed framework to test and verify the effectiveness of wearable safety promotion devices, which will be the focus of future efforts.

## 5. Conclusions

This paper presents a review of various on-body placements that can influence the acceptance of wireless communication from a field personnel’s safety viewpoint. The successful application of communicating safety status on construction sites requires not only technological advances but also the practicality of the wearable devices. Seven unique on-body placements, responsible for auditory, visual and tactile communication, have been identified and evaluated for their ability to convey meaningful information. They were hard hat, eye glasses, safety vest, arm band, wrist band, waist belt and thigh pad. Additional placement possibilities were also discussed regarding the attachment of portable safety devices such as knee pad and shoe soles. Among the various on-body placements discussed, the hard hat was the most favored placement by researchers.

Additional insights from the literature, for the successful application of wearable wireless safety communication on construction sites, can be narrowed down to the following themes.

1. Wearable auditory communication devices should be able to overcome the background noise on a construction site and have been predominantly placed on the hard hat for its proximity to the ears.
2. While research in wearable visual communication devices is limited, hard hat and wrist band have been the preferred on-body placements for information about workplace hazards. Eye glasses, with the ability to overlay safety information on the field of view, have also received consideration.
3. The ability to use wearable tactile communication to convey safety information has also been explored with the placement of vibration motors on hard hat, safety vest, and waist belt being favored over other on-body placements.
4. Two previous studies made efforts to test auditory, visual and tactile communication on a single on-body placement, the safety vest, and the wrist band.
5. 15 prior studies made efforts to convey rich wearable safety alerts as opposed to static alerts signifying mere presence or absence of danger. These were spread over six different on-body placements.
6. A majority of the attempts to convey rich wearable safety alerts (8 out of 15) involved communication through visual mode.
7. Furthermore, a novel communication network is presented to visualize the generation of wearable safety alerts for each mode of communication, and insights on future research and development are offered.

Considering the limited number of papers available about the on-body placement of wearable safety promotion devices, their relation to the sensory communication modalities, and the associated response of the wearer, it is not logical to generalize the results. However, the limitations and potential discussed here are expected to be valuable resources to consider when developing and implementing wearable communication devices suitable for construction sites. Continuation of this research is prudent and additional studies should be carried out to determine to the optimal on-body placement of wearable safety devices, and the factors influencing such decision.

## Figures and Tables

**Table 1:** Selected literature and corresponding source of publication.

Publication	Number of Papers	References
Automation in Construction	7	[111,112,113,114,115,116,117]
Sensors	6	[118,119,120,121,122,123]
Safety Science	2	[124,125]
International Pervasive and Ubiquitous Computing and International Symposium on Wearable Computers	2	[126,127]
International Health and Safety Conference	1	[128]
Commercial Vehicle Engineering Congress and Exhibition	1	[129]
International Conference on Mobile and Ubiquitous Multimedia	1	[130]
International Conference on RFID	1	[131]
International Journal of Artificial Intelligence	1	[132]
International Journal of Environmental Research and Public Health	1	[133]
International Symposium on Automation and Robotics in Construction	1	[134]
International Symposium on Robotic and Sensor Environment	1	[135]
Journal of Computer Communications	1	[136]
Journal of Computing in Civil Engineering	1	[137]
Journal of Construction Engineering and Management	1	[138]
Journal of Sensors	1	[139]
	29	

**Table 2:** Simplified node definitions for the novel communication network.

Node	Representation
Satellite/Cellular Station	A satellite or local cellular base station
Cloud Server	A central processing unit interacting with multiple nodes wirelessly
Human Wearable	An active or passive portable device worn by the jobsite personnel
Movable Machine	A human-operated or automated machine with active movement
Construction Environment	Any stationary beacon, tag, reader or access point on the jobsite
Wireless Proof of Concept	Wireless communication/assessment without localization (positioning) attempt

**Table 3:** The review criteria resulted in 29 unique research papers. A majority of these papers utilized auditory alarms 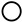 (19), followed by tactile feedback 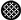 (15), and visual alerts 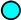 (12). Hard hat, with eleven instances, was the most frequent on-body placement. The studies conveying information beyond a binary presence or absence of hazard are recognized with a star mark - 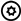, 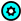, and 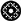, for auditory, visual, and tactile communication, respectively.

Reference	Year	Hard Hat	Safety Vest	Waist Belt	Wrist Band	Eye Glasses	Thigh Pad	Arm Band
[111]	2005	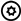						
[129]	2007			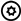 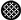				
[117]	2010							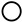 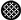
[112]	2011	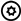						
[131]	2011	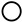						
[136]	2012		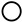					
[137]	2012	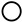						
[113]	2014			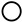 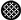				
[135]	2014	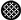						
[128]	2015		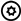 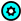					
[126]	2015		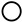 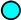 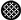					
[130]	2015				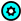			
[125]	2016	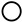 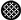						
[138]	2016						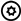 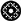	
[134]	2016						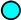	
[139]	2016				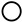 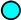			
[116]	2017					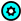		
[120]	2018		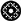					
[124]	2018	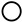 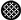						
[115]	2018				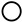 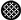			
[121]	2019	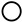 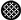						
[123]	2019			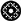				
[127]	2020	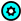 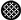						
[133]	2020					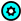		
[118]	2020	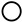 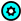						
[119]	2020	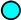						
[132]	2020			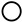 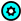				
[114]	2021		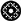					
[122]	2021				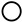 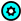 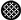			
		11	5	4	4	2	2	1
